# Editorial: Thinking and doing intersectionality in sociology of sport

**DOI:** 10.3389/fspor.2023.1212457

**Published:** 2023-05-18

**Authors:** Madeleine Pape, Lucie Schoch, Akilah Carter-Francique

**Affiliations:** ^1^ Institute of Sports Sciences, Université de Lausanne, Lausanne, Switzerland; ^2^ School of Education, Health, and Human Services, Benedict College, Columbia, MO, United States

**Keywords:** intersectionality, gender, feminism, critical theory, activism, policy, physical activity, health

**Editorial on the Research Topic** Thinking and doing intersectionality in sociology of sport

Since the field of sport sociology was formalized in the 1960s, starting with the International Committee for the Sociology of Sport in 1965 (now ISSA), proponents of the field have sought to “promote, stimulate, and encourage the sociological study of play, games, and contemporary physical culture” (1). Across diverse national contexts, sociological research has sought to understand the legislation, advocacy, and activism needed to create equitable and inclusive sporting structures and practices. Arguably, these efforts have had meaningful impacts: for example, in 2016, the International Olympic Committee (IOC) recognized widespread sexual and psychological harassment and abuse in sport (2), reflecting decades of work by feminist scholars to compel decision-makers to take action (3). Much of this research has highlighted the role of gender, sexuality, racialization, nation, disability, and class as systems of difference-making and hierarchy that generate inequalities in and through sport.

Less attention is given to how these systems intersect in sporting contexts, with consequences for whose experiences of oppression are made visible and become worthy of sociological inquiry, despite sport demonstrably lending itself to intersectional analysis (4, 5). Consider, for example, the unspoken Whiteness of the standard swim cap, with a swim cap for Black hair rejected by the international governing body for swimming as not fitting “the natural form of the head” (6).

We came together with the desire to amplify the concept of intersectionality and how it serves as a tool to understand and redress social inequities in sport. We are self-identified scholar advocates that use our scholarship to expose inequities and amplify practices to promote social change. We have seen in both our work and lived experiences how intersectional approaches are necessary to explain and address the forms of inequality, exclusion, and violence that continue to mark the sporting experiences of many people.

The concept of intersectionality has become a defining paradigm for critical scholarship, growing out of the legacy of Black feminist thought and efforts to hold White institutions, White feminists, and civil rights movements to account for obscuring the experiences and voices of Black women (7). At its core, the concept challenges a focus on singular categories (e.g., women) or systems of difference-making (e.g., gender) as rendering invisible those who find themselves multiply marginalized (e.g., women with disabilities). Far more than a theory of individual identity, intersectionality conceptualizes the structure of social life as a “matrix of domination,” in which systems of difference-making and inequality are always co-present and mutually constitutive (8).

Yet, questions about the relevance and utility of intersectionality remain: has it become a “buzzword” devoid of critical content (9), divorced from its Black feminist roots and appropriated by White feminisms (10)? (How) can it be translated from theory to a mode of inquiry and practice, given the complexities of operationalizing simultaneous systems of difference-making and oppression (11, 12)? In the wake of the COVID-19 pandemic, the intensification of protest related to racialized and gendered injustices, and growing institutional attacks on critical scholarship and teaching across numerous countries, the moment is ripe to reflect on the concept of intersectionality and its relationship to sport sociology.

We are excited to share eight works from scholars that employ an intersectional lens to examine the reproduction of difference and inequality in and through sport. Two articles offer the perspective of “outsiders within,” highlighting the voices of women of color in the United States and Global South. Ajhanai Channel Inez Keaton examines how five Black women who are Athletic Diversity and Inclusion Officers at US universities perceive organizational inclusivity. Keaton shows how these women translate their positionality as “outsiders within” predominantly White departments into a form of expertise, which they use to challenge conditions of intersectional marginalization. Nana Akua Achiaa Adom-Aboagye writes from her positionality as a Black African feminist scholar, reviewing the sociological literature on women coaches to show how the experiences of women in Africa are typically absent. So, too, are African scholars missing among those who are frequently cited in relation to coaching. Adom-Aboagye calls on Global North scholars to look to the African continent as a space of original knowledge production, insights which are necessary to have a more complete understanding of women's coaching experiences.

Three further articles also consider understudied intersections in sport sociology. Laurent Paccaud examines the co-conditioning of dis/ability and gender, using an ethnographic study of powerchair hockey to create a powerful account of the need to study the margins in order to render visible the hidden workings of gender relations and inequality in sport. Paccaud's article shows how gender ideology can be reproduced even in the absence of (assumed) gender differences in sporting ability, while allowing powerchair hockey to tell its own story as a study site in its own right. Griffin et al. offer a social media analysis of the body positivity movement, illustrating how it has become divorced from radical forms of resistance, co-opted by privileged women, and transformed into a neoliberal, gentrified, and cis-heteronormative tool for reproducing the worthy (White, able-bodied, “fit”) body. An article by Reynolds et al. focuses on spectator behavior and youth sport, showing how gender and race mutually shape the actions of parents in youth sport settings.

Three articles consider alternative structures and spaces of resistance. Symons et al. offer a case study of the Outer Sanctum podcast, showing how this Australian sports media platform has increased the profile of underrepresented voices, thereby contributing to making Australian media coverage of sport more intersectional in its representation and content. Bell et al. consider whether and how online fitness platforms could offer LGBTQ2S+ people alternative spaces for creating community and engaging in physical activity. They suggest that in addition to building intentional communities for the LGBTQ2S+ communities, education programming is needed to ensure that coaches, fitness trainers, and owners provide safe and inclusive spaces for diverse patrons. Emma Calow reflects on how the sporting field and the sociology of sport classrooms can serve as spaces of protest and transformation. Calow argues that athlete activism is always already intersectional and that the sociological classroom is always already a space of social justice action, with both offering allies the opportunity to advance intersectional causes.

Combined, we hope that the contributions to this research topic will support reflection and discussion in the sociology of sport community on the role of intersectionality and how it can serve as a tool to understand and redress social inequities in sport.
